# Non‐bacterial cystitis with increased expression of programmed death‐ligand 1 in the urothelium: An unusual immune‐related adverse event during treatment with pembrolizumab for lung adenocarcinoma

**DOI:** 10.1002/iju5.12211

**Published:** 2020-08-12

**Authors:** Yohei Ueki, Masahiro Matsuki, Terufumi Kubo, Rena Morita, Yoshihiko Hirohashi, Syunsuke Sato, Ryota Horibe, Kazuhiko Matsuo, Tomohide Tsukahara, Takayuki Kanaseki, Yasunari Takakuwa, Masaaki Satoh, Naoki Itoh, Toshihiko Torigoe

**Affiliations:** ^1^ Department of Urology NTT‐East Corporation Sapporo Medical Center Sapporo Japan; ^2^ Department of Pathology School of Medicine Sapporo Medical University Sapporo Japan; ^3^ Department of Respiratory Medicine NTT‐East Corporation Sapporo Medical Center Sapporo Japan; ^4^ Sapporo Clinical Laboratory Inc. Sapporo Japan; ^5^ Department of Clinical Pathology NTT‐East Corporation Sapporo Medical Center Sapporo Hokkaido Japan

**Keywords:** cystitis, immune checkpoint inhibitor, immune‐related adverse event, PD‐L1

## Abstract

**Introduction:**

Immune checkpoint inhibitors are now a standard therapeutic option for lung adenocarcinoma. However, Immune checkpoint inhibitors often induce various immune‐related adverse events.

**Case presentation:**

The patient was a 78‐year‐old woman with lung adenocarcinoma who had a partial response to pembrolizumab. During treatment, she complained of pollakiuria and nocturia with painful micturition. Histological analysis revealed infiltration of CD8‐positive and/or TIA‐1 cytotoxic granule‐associated RNA binding protein‐positive lymphocytes and programmed death‐ligand 1 expression in the urothelium. A diagnosis of immune‐related adverse event cystitis was made based on these clinical and pathological findings. The patient’s subjective symptoms and findings on cystoscopy improved dramatically after treatment with prednisolone.

**Conclusion:**

Immune checkpoint inhibitors‐induced cystitis is extremely rare. This report is the first to include an immunohistochemical analysis of the urothelial epithelium in immune‐related adverse event cystitis and describes an instructive case.

**Abbreviations & Acronyms**
ICI: immune checkpoint inhibitor
IFN‐γ: interferon‐gamma
irAE: immune‐related adverse event
PD‐L1: programmed death‐ligand 1
SCC: squamous cell carcinoma

**Keynote message**

This report describes a patient with adenocarcinoma of the lung who showed a partial response to pembrolizumab but complained of dysuria. Histologically, we found infiltration of CD8‐ and/or cytotoxic granule‐positive lymphocytes, and expression of PD‐L1 in the urothelium. We made a diagnosis of cystitis as an irAE associated with pembrolizumab, which is extremely rare.

## Introduction

ICIs have dramatic therapeutic effects and are now established as standard therapy for many types of cancer. However, ICIs cannot induce cancer‐specific immunity, and ICIs often provoke autoimmune‐mediated unfavorable effect, namely irAEs, of which reports are increasing. A correlation has recently been demonstrated between the clinical effects of ICIs and occurrence of irAEs.^1^, ^2^ Therefore, immunotherapy can be considered to be a double‐edged sword. Various irAEs can occur depending on the site blocked by the antibody.^3^ However, urogenital irAEs are rare with the ICIs presently in clinical use. Here we describe a patient who developed irAE cystitis after administration of pembrolizumab for lung adenocarcinoma and was found to have increased expression of PD‐L1 in the urothelium. This is the first report of such a case.

## Case presentation

**Fig. 1:**
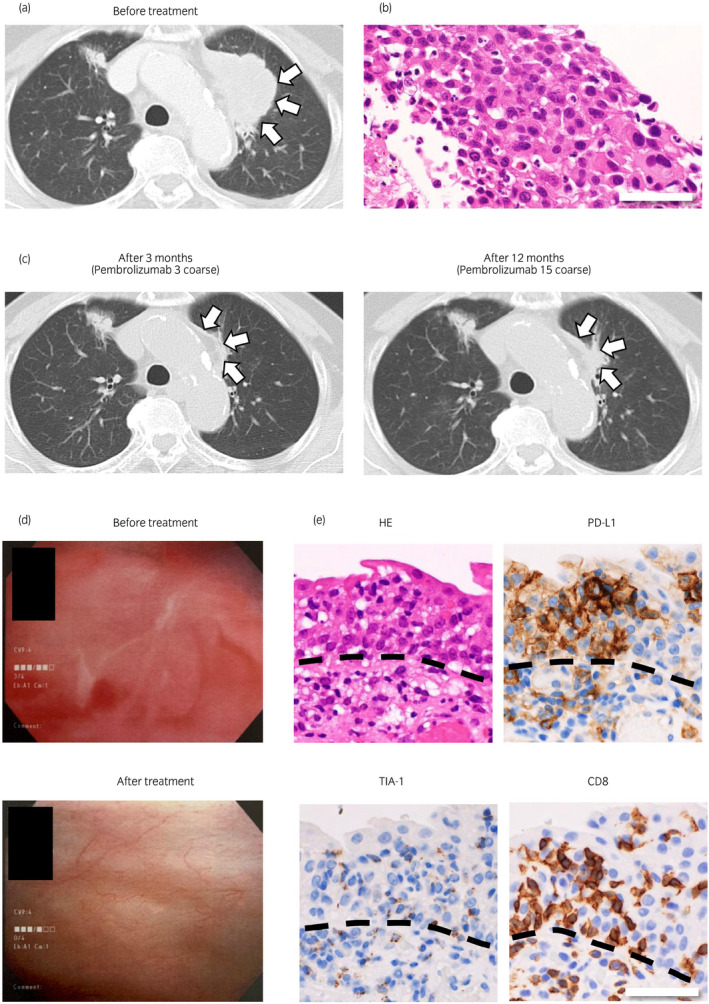
Clinical data and pathological findings. (a) A computed tomography image shows a space‐occupying lesion measuring 58 × 32 mm in the S3 segment of the left lung (arrows). (b) A microscopic image shows a nest‐like proliferation of tumor cells containing enlarged rounded nuclei with eosinophilic cytoplasm (hematoxylin and eosin staining; original magnification ×200; bar = 50 μm). (c) After 3 (left panel) and 12 (right panel) doses of pembrolizumab, the lesion became smaller (arrows). (d) A cystoscopy image obtained before (upper panel) and after (lower panel) administration of prednisolone. (e) Histopathological image of the urinary bladder. The urothelium strongly expressed PD‐L1 but did not show significant atypia suggesting malignancy. PD‐L1‐positive cells were also found in the subepithelial tissue. These cells were presumed to be histiocytes. Infiltrates of CD8‐positive and/or TIA‐1‐positive lymphocytes are present in the epithelium. Dotted lines indicate the epithelial‐subepithelial margin (original magnification ×200; bar = 50 μm).

A 78‐year‐old woman with no smoking history presented to our hospital with a complaint of epigastric distress. Computed tomography identified a 58 × 32‐mm space‐occupying lesion suggestive of lung cancer in the left upper lobe (Fig. 1a, arrows). The mass lesion involved the mediastinum, and metastases to the lymph nodes on the contralateral side of the mediastinum and to the right lung (21 × 11‐mm) were suspected. Histological analysis showed a nest‐like proliferation of tumor cells that contained enlarged and rounded nuclei and eosinophilic cytoplasm (Fig. 1b). Immunohistochemical analysis revealed that the tumor was positive for thyroid transcription factor‐1 but negative for ΔNp63 (p40), indicating adenocarcinoma of the lung (data not shown). The tumor cells expressed PD‐L1 (clone 22C3; Dako, Glostrup, Denmark) at a rate of more than 95% (data not shown). The lung mass did not contain anaplastic lymphoma kinase or c‐ros oncogene 1 receptor tyrosine kinase fusion proteins and harbored epidermal growth factor receptor exon 20 insertions. The tumor was evaluated as cT4bN3M1a based on the TNM Classification of Malignant Tumors 8th edition (Union for International Cancer Control, Geneva, Switzerland). The patient received 17 cycles of 200‐mg doses of pembrolizumab. She developed slight interstitial pneumonia and a mild increase in HbA_1c_ during the first four cycles. While these might be irAE, both symptoms had been improved without additional intervention. A partial response to pembrolizumab according to the Response Evaluation Criteria in Solid Tumors guidelines was found at 3 months (after three cycles) and 12 months (after 15 doses; Fig. 1b). After six cycles of treatment, the patient complained of pollakiuria and nocturia accompanied by painful micturition. These symptoms had been present for 6 months. Urine culture did not identify a causative microorganism, and administration of a β3‐adrenergic receptor agonist did not improve the symptoms. Urinalysis showed red blood cells >100/HPF and white blood cells >100/HPF. Urine culture did not detect any causative organisms. There was no atypical cell which is suspected of malignant tumor in the three times of urine cytology. Cystoscopy showed that the entire bladder mucosa was red and edematous but there was no evidence of tumor (Fig. 1d, upper panel). This was similar to common cystitis. For excluding malignancy, we performed a cold punched bladder biopsy to obtain the pathological findings. Histological analysis revealed numerous infiltrates of CD8‐positive cells and/or TIA‐1 (TIA‐1 cytotoxic granule‐associated RNA binding protein)‐positive lymphocytes into the urothelium but no atypia suggesting malignancy (Fig. 1e). Interestingly, the urothelial epithelium infiltrated by lymphocytes and subepithelial inflammatory cells strongly expressed cell boundary PD‐L1 (clone E1L3N; Cell Signaling Technology, Danvers, MA, USA) (Fig. 1e). Small biopsy tissue could not provide definite relationship between CD8‐positive cell infiltration and PD‐L1 expression in the urothelium. However, at least, urothelial epithelium without lymphocytic infiltration did not express PD‐L1. Based on these clinical and pathological findings, we made a diagnosis of irAE cystitis. The patient’s subjective symptoms and findings on cystoscopy improved dramatically after 19 days of treatment with prednisolone 25 mg/day (Fig. 1d, lower panel). The prednisolone was tapered and stopped after 2 months. No further pembrolizumab was administered after starting prednisolone and there was no recurrence of dysuria.

## Discussion

Pembrolizumab was clinically effective for adenocarcinoma of the lung in this case. Recent reports have shown a relationship between the clinical effect of ICIs and the frequency of irAEs in the gastrointestinal and endocrine organs but not in the urogenital tract.^1^, ^2^ However, accumulating evidence indicates that irAEs can occur in any organ or tissue. Interestingly, the urothelial epithelium in our patient, despite an absence of malignancy, showed strong expression of PD‐L1, an IFN‐γ‐inducible molecule.^4^ Furthermore, the numerous lymphocyte infiltrates characteristically showed high TIA‐1 and/or CD8 expression. This finding suggests that the infiltrating lymphocytes had potent cytotoxic activity and produced IFN‐γ, meaning that they were able to attack the urothelial epithelium. PD‐L1 expression in the urothelium was considered to be the response to IFN‐γ. After excluding any other differential diagnosis, these histological findings convinced us that the dysuria of the patient was an irAE. Although overactivation of the immune system is clearly the underlying cause of irAEs, the precise mechanism involved remains unclear. Our case raises the possibility that the unknown antigen in the urothelium is targeted by lymphocytes that are positive for TIA‐1 and/or CD8.

It is important to be able to recognize an irAE regardless of the mechanism. ICI‐induced cystitis is extremely rare, with only three cases reported in the literature so far (Table 1).^5^, ^6^ In the clinical setting, a complaint of dysuria from a patient on ICI therapy should be considered a diagnostic clue for irAE cystitis. This is the first report of expression of PD‐L1 in the urothelial epithelium in a patient with irAE cystitis. Although not shown here, we have performed immunohistochemical studies to look for expression of these molecules in a few cases of cystitis of unknown cause. Although the lesions in those cases also harbored CD8‐positive and TIA‐1‐positive lymphocytes at levels similar to those in the patient in this case, we did not detect the expression of PD‐L1 in urothelial epithelium in the patients with cystitis of unknown cause (data not shown). Performing immunohistochemistry for PD‐L1 in a larger number of cases might be useful for distinguishing irAE cystitis from other types of cystitis.

**Table 1:** Clinical information of the case reports of irAE cystitis.

Case	Symptoms	Objective findings	Primary disease	ICIs (cycles)	Treatment	ICIs after irAE cystitis	Pathological features	Reference
50 y, male	Micturition pain, pollakisuri	Pyuria negative urine culture	Lung SCC	Nivolumab (7)	Prednisolone (1 mg/kg/day)	Discontinue because readministration of nivolumab reactivate symptoms	None	5
60 y, male	Micturition pain, pollakisuria	Pyuria negative urine culture	Lung SCC	Nivolumab (12)	Discontinue ICIs	Discontinue	None	5
62 y, male	Micturition pain, pollakisuria	Microhematuria and pyuria Cystoscopy: diffuse redness and erosion Urine cytology: negative	Lung SCC	Nivolumab (4)	Methylprednisolone 500 mg x 3 days and prednisolone (0.5 mg/kg/day)	Continue with concomitant prednisolone	Epithelial desquamation and edematous changes	6
78 y, female	Micturition pain, pollakisuria	Microhematuria and pyuria Cystoscopy: diffuse redness and erosion Urine cytology: negative	Lung adenocarcinoma	Pembrolizumab (6)	Prednisolone (0.5 mg/kg/day) and discontinuing ICIs	Discontinue	PD‐L1+ urothelial cells and CD8+ and/or TIA‐1+ infiltrating lymphocytes	This case

In conclusion, the expanding indications for ICIs have been associated with a rapid increase in reports of irAEs, which have included a range of clinical signs in various organs. Although our patient’s cystitis was not life‐threatening, her dysuria, which included pollakiuria, nocturia, and painful micturition, had a significant impact on her quality of life. This is an instructive case for oncologists and other specialists who manage irAEs. The possibility of an irAE should be considered when a patient has complaints after ICIs administration.

## Conflict of interest

K. Matsuo is an employee of Sapporo Clinical Laboratory Inc. The other authors have no conflict of interest in regard to this report.
